# Hepatitis B Virus Genotypes and Antiviral Resistance Mutations in Romanian HIV-HBV Co-Infected Patients

**DOI:** 10.3390/medicina58040531

**Published:** 2022-04-11

**Authors:** Camelia Sultana, Mihnea Casian, Cristiana Oprea, Irina Ianache, Camelia Grancea, Daniela Chiriac, Simona Ruta

**Affiliations:** 1 Faculty of Medicine, Carol Davila University of Medicine and Pharmacy, 050474 Bucharest, Romania; mihnea.casian@gmail.com (M.C.); cristiana.oprea@spitalulbabes.ro (C.O.); simona.ruta@umfcd.ro (S.R.); 2 Emergent Diseases Department, Stefan S. Nicolau Institute of Virology, 030304 Bucharest, Romania; cgrancea@yahoo.co.uk (C.G.); dana.chiriac1976@gmail.com (D.C.); 3 Cardiology Department II, Dr Carol Davila Central Military Emergency Hospital, 010825 Bucharest, Romania; 4 HIV/SIDA Department I, Dr Victor Babes Hospital of Infectious and Tropical Diseases, 030303 Bucharest, Romania; ianacheirina@yahoo.ca

**Keywords:** HIV–HBV co-infection, Romania, HBV genotypes, resistance-associated mutations, HIV pediatric cohort, injecting drug users

## Abstract

*Background and Objectives*: Romania has one of the highest prevalence of hepatitis B virus (HBV) infection in human immunodeficiency virus (HIV) patients, mostly in those parenterally infected during childhood; nevertheless, there are scarce data on the virological profile of co-infection. The objective of this study was to assess the prevalence of HBV genotypes and antiviral resistance-associated mutations (RAMs) in these co-infected patients, in order to monitor the viral factors associated with the evolution of liver disease. *Materials and Methods*: HBV genotypes and RAMs were detected using nested PCR and line probe assays (INNO-LiPA HBV genotyping assay, and INNO-LiPA HBV DR v2, Innogenetics). *Results*: Out of 117 co-infected patients, 73.5% had detectable HBV-DNA, but only 38.5% presented an HBV viral load >1000 IU/mL. HBV genotype A was present in 66.7% of the cases and was dominant in patients parenterally infected during early childhood, who experienced multiple treatment regimens, with a mean therapy length of 15.25 years, and present numerous mutations associated with lamivudine (LAM) resistance, but very rarely active liver disease. HBV genotype D was detected in 33.3% of the cases, mostly in recently diagnosed injecting drug users who are treatment naïve, but, nevertheless, present RAMs in 63.5% of the cases, suggesting transmitted drug resistance, and display more frequently advanced liver fibrosis (36.1% vs. 12.3%; *p* = 0.033). The most frequently encountered RAMs are M204V/I: 48.8%, L180M: 33.3%, L80V: 28.8%, and V173L: 42.2%. There are no significant differences in the distribution of RAMs in patients infected with different HBV genotypes, except for the L80V and N236T mutations, which were more frequently found in HBV genotype A infections (*p* = 0.032 and *p* = 0.004, respectively). *Conclusions*: HBV genotypes A and D are the only genotypes present in HIV–HBV co-infected patients from Romania, with different distributions according to the infection route, and are frequently associated with multiple RAMs, conferring extensive resistance to LAM.

## 1. Introduction

Co-infection with hepatitis B virus (HBV) is common in human immunodeficiency virus (HIV) infected individuals, due to them having similar risk factors and routes of transmission (parenteral, sexual, and perinatal), and represents an important cause of non-AIDS-related mortality [1]. Worldwide, it is estimated that 5–20% of the people living with HIV are also chronically infected with HBV [2]. Mapping the HIV–HBV co-infection among specific risk groups is hard to achieve, as important data regarding large geographical regions are still missing. The highest prevalence rates of HIV–HBV co-infection are reported among injecting drug users (IDUs), especially in Latin America, South and East Asia, and East Africa (27.3%, 16.3%, 15.4% and 13.9%, respectively) [3]. On the contrary, in Europe, the prevalence of HIV–HBV co-infection among the IDU population is reported to be lower, ranging between 0.1 and 11% [3,4], probably due to the higher accessibility of needle exchange programs, voluntary counseling and testing, and other harm reduction measures, all indispensable for the successful prevention of blood-borne infections. In Romania, during a major pediatric HIV epidemic, acquired parenterally in the late 1980s, the prevalence of HBV co-infection was extremely high [5,6,7]. Twenty years later, almost half of the long-term survivors remain chronic HBV carriers, and maintain active HBV replication, but rarely display signs of liver disease progression, probably due to prolonged immunotolerance [8]. Nonetheless, after the introduction of universal HBV vaccinations for newborns in this country in 1995, the incidence of HBV infections in HIV-infected persons decreased continuously [9], until 2011, when there was a major outbreak of blood-borne viral infections in IDUs [10]. During the past years, in Romania, the prevalence of HBV infection in newly diagnosed HIV-infected patients decreased from 12.5% in 2018 to 7.3% in 2020 [10].

High rates of liver morbidity and a high incidence of hepatocellular carcinoma (HCC) are reported in HIV–HBV co-infected patients, a consequence of HIV-induced immune deficiency, HBV replication, and immune-mediated hepatocytolysis [1,11]. HBV genotypes can also influence the progression of liver disease. Out of the ten HBV genotypes (A–J), with each being defined as >8% nucleotide differences in the whole genome sequence and >4% in the *S* gene [12], genotype A is linked to an increased risk of progression to chronic infection in adults, and genotype D to acute liver failure [13], while genotype C is associated with high rates of progression to cirrhosis and hepatocellular carcinoma, probably due to its association with higher viral loads and core/precore genomic mutations [11,14]. Scarce information about the distribution of HBV genotypes is available for Romania, with genotypes A, D and, rarely, F being detected in HBV mono-infected individuals [15,16], and no data on the circulating genotypes in HIV co-infected patients. 

The objective of this study is to assess the prevalence of HBV genotypes and their association with liver disease progression and resistance-associated mutations (RAMs) in HIV–HBV co-infected patients admitted to a tertiary facility in Bucharest, Romania. 

## 2. Materials and Methods

### 2.1. Serum Samples

A retrospective, cross-sectional study was conducted on stored samples collected between 2010 and 2018 from HIV–HBV infected patients followed up in “Victor Babes” Clinical Hospital for Infectious and Tropical Diseases, Bucharest. Sera were tested in the Stefan S. Nicolau Institute of Virology, Bucharest, for serological and molecular markers of viral hepatitis B, including anti-HBc IgG antibodies (markers of past or present HBV infection), HBsAg (marker of chronic HBV carriage), HBeAg and HBV-DNA (markers of active viral replication), and HDV antibodies-markers of hepatitis D virus (HDV) co- or super-infection. All HBV chronic co-infected patients were included in the study; patients positive for serological markers of hepatitis C viral infection were excluded.

The study protocol was approved by the Ethics Committee of the Stefan S. Nicolau Institute of Virology, in accordance with the 1975 Declaration in Helsinki. Informed consent was obtained from each patient.

### 2.2. Detection and Quantification of Serum HBV-DNA

HBV-DNA was isolated from venous blood samples using a QIAamp DNA blood mini kit (Qiagen GmbH; Hilden, Germany) and HBV viremia was measured by real-time PCR (COBAS TaqMan HBV Test; Roche Molecular Systems, Branchburg, NJ, USA), with a linear range for viral load quantification of 20 to 1.7 × 10^8^ IU/mL.

### 2.3. Detection of HBV Genotypes 

HBV genotypes were detected using a line probe assay (INNO-LiPA HBV Genotyping assay; Innogenetics N.V., Ghent, Belgium), after HBV polymerase (gene domain B to C) amplification by nested PCR. The amplified HBV genomic region extends between nucleotides 415 and 824 for the outer primers, and nucleotides 456 and 798 for the nested inner primers, and identifies hepatitis B virus genotypes A to H.

### 2.4. Detection of HBV Resistance-Associated Mutations (RAMs)

HBV RAMs were detected using a line probe assay (INNO-LiPA HBV DR v2, Innogenetics N.V., Ghent, Belgium), by reverse hybridization of the 867 bp amplified HBV product to specific oligonucleotide probes coated onto strips, allowing the detection of HBV Pol gene mutations and the wild-type genome.

### 2.5. Quantification of Serum HIV-RNA

The HIV viral load was determined using a commercial nucleic acid amplification test (COBAS TaqMan HIV-1 Test Version 2.0, Roche Molecular Systems, Branchburg, NJ, USA), with a linear range between 34 and 10,000,000 copies of HIV RNA/mL.

### 2.6. Assessing of the Immunologic Status

The CD4 cell number was determined using a flow cytometry-based assay (BD Tritest CD4/CD8/CD3, Becton Dickinson, San Jose, CA, USA).

### 2.7. APRI Score (AST-to-Platelet Ratio Index) 

The APRI score (AST-to-Platelet Ratio Index) was used as a non-invasive serological marker with a high predictive value for advanced liver fibrosis and cirrhosis, calculated as [(AST/upper limit of the normal AST range) × 100]/Platelet Count. An APRI score greater than 1.0 has a sensitivity of 76% and a specificity of 72% for predicting cirrhosis [17].

### 2.8. Statistical Analysis 

The statistical analysis was conducted with SPSS v.23 (Chicago, IL, USA), using Pearson correlation test for direct correlation of the quantitative variables, and T test to compare means and variance for the variables in different risk groups. Contingency tables and the chi-squared test were used to verify the independence between the parameters. *p* values below 0.05 were considered statistically significant.

## 3. Results

### 3.1. Patients Characteristics

The study included 117 HBV–HIV co-infected patients (mean age 29.7 years (19–43), 64.9% males), belonging to the following two important risk groups for the acquisition of both infections: long-term survivor patients from the Romanian cohort parenterally infected with HIV during early childhood (69.2%), and newly diagnosed injecting drug users (IDUs, 30.8%). HIV–HBV co-infected long-term survivors had a mean duration of HIV infection of 27.03 years, and a similar duration of HBV infection, and experienced multiple treatments, with a mean length of antiretroviral therapy of 15.25 years. In contrast, IDUs (with a declared mean length of injecting drugs of 10.72 years) were recently diagnosed, with an unknown duration of HIV and HBV infections, and were untreated. The patients’ characteristics are presented in Table 1.

Undetectable HIV and HBV viral loads were more frequently observed in patients from the long-term survivors cohort; in those with active replication, the mean HIV-RNA viral load was significantly lower than in IDUs (4 log10 copies/mL vs. 5.5 log10 copies/mL), in which no differences were detected for the mean HBV viral load. The APRI score was significantly higher in IDUs; most of them had values greater than 1.0, predictive of severe liver fibrosis, as compared with the long-term survivors, who showed no signs of advanced liver disease (APRI = 4.9 ± 2.189 vs. 0.57 ± 0.092; *p* = 0.004).

### 3.2. Treatment Regimens

At the time of the study, 76 out of 81 (93.8%) HIV–HBV co-infected patients with long-term infection were treated for HIV infection, with 61.7% of them being treated for more than 10 years, and the rest abandoning treatment due to therapeutic fatigue. All the therapeutical combinations included nucleoside reverse transcriptase inhibitors (NRTI); the current regimen contained a combination of two NRTIs and protease inhibitors (PIs) in 76.5% of the patients, while the rest were treated with a two NRTI/NNRTI-based regimen or other individualized regimens.

A total of 75.3% of the patients were treated with a regimen, including dual HIV–HBV-active antiretroviral therapy. Currently, all co-infected patients are receiving a dually active antiretroviral regime, including tenofovir (TDF/TAF) + lamivudine (LAM), or TDF/TAF + emtricitabine. A third of the long-term treated patients (26/76, 34.2%) had self-reported treatment adherence problems. All the injecting drug users were treatment naïve.

### 3.3. HBV Genotypes in HIV–HBV Co-Infected Patients

HBV-DNA was detectable in 86 of the HIV–HBV co-infected patients (73.5%), but only 45 had an HBV viral load >1000 IU/mL, and qualified for HBV genotyping (26 from the long-term survivors cohort and 19 from the drug users group). Among them, genotype A and genotype D were detected in 66.7% and 33.3% of the cases, respectively. Overall, there were no significant differences in the HIV or HBV virological parameters, nor in the presence of advanced liver fibrosis between the HBV genotypes, as shown in Table 2. HBV genotype A was dominant in the long-term survivors from the HIV pediatric cohort (*p* = 0.003), correlated with transfusions and parenteral procedures in the past (*p* = 0.004), while genotype D was predominant in the IDUs (*p* = 0.003).

### 3.4. Antiviral Resistance-Associated Mutations (RAMs)

The HBV wild-type genome was present in only seven samples (15.5%), all from the untreated IDU patients, while the rest had one or multiple RAMs (one sample presented nine RAMs). All patients from the long-term infected cohort and 63.2% of the IDUs harbored RAMs. 

There were no significant differences in the distribution of RAMs in patients infected with different HBV genotypes, except for the compensatory L80V mutation and the adefovir (ADV) resistance mutation N236T, which were more frequently found in HBV genotype A infections (*p* = 0.032 and *p* = 0.004, respectively), as shown in Table 3.

The most frequently encountered profiles of amino acid substitutions, conferring resistance to different nucleotide analogues [18] and their combinations, are presented in Table 4, and are as follows:

- M204V/I: 48.8%, plus L180M: 33.3% ± L80V: 28.8% and V173L: 42.2%—a profile suggestive for LAM resistance (with all mutations, except for M204V, also being associated with telbivudine resistance, and the combination of L180M, M204V, andV173L being associated with entecavir (ETV) resistance.
- N236T: 28.8% and A181T/V: 15.5%—a profile suggestive for ADV resistance (and associated with reduced susceptibility to TDF/TAF). No specific RAM combinations were observed, according to the infecting genotype.

Advanced liver fibrosis (APRI >1) was only present in patients harboring RAMs (15.7% vs. 0% in patients with wild-type), although the HBV viral load tended to be lower in these patients, compared to those infected with wild-type viruses (6.48 log10 vs. 6.9 log10; *p* = 0.015).

## 4. Discussion

This is, to the best of our knowledge, the first study to investigate the prevalence and distribution of HBV genotypes and resistance-associated mutations in HIV–HBV co-infected patients from Romania. Here, we report the presence of HBV genotype A, mainly in the cohort of HIV–HBV long-term survivors infected during early childhood, with the presence of extensive resistance to LAM. LAM was the most frequent NRTI used in long-term cART regimens in developing countries [19], including Romania; its rapid selection of HBV-resistant strains is well documented, even in mono-infected patients, with RAMs being present in more than 50% of treated patients after 2 years, and in more than 90% of those treated for more than 4 years [20]. Therapeutic fatigue and the current lack of treatment adherence might explain the high percentage of RAMs in the study patients. The low number of genotyped samples is a limitation of our study, justified by the fact that a significant number of the patients (the long-term survivors infected during their early childhood) are now effectively treated with dually active therapy (TDF/TAF + LAM or TDF + FTC), and have undetectable HBV viral loads. Nevertheless, the study included the majority of HIV–HBV infected patients followed up in one of the largest hospitals for infectious diseases in Romania; as such, it may offer a hint about the country’s epidemiological situation.

There is no statistically significant difference between genotypes in terms of RAM association. The most frequently LAM-associated mutations detected in this study were M204V and V173L, commonly reported worldwide across all genotypes, with a similar distribution in HBV genotype A and D infections, as dual (M204V + L180M) and triple mutations (V173L + L180M + M204V). Comparable results were reported by previous studies, although a trend towards more frequent RAMs among patients with HBV genotype A infections, as compared with HBV genotype D, was suggested [21,22]. Their presence has been associated with numerous immune-escape mutations in preS/S ORF, due to partial overlapping of the S and Pol genes [23,24,25,26]; recent reports in extensively treated HIV–HBV co-infected patients in Europe indicate that genotype A increases the selection of these NRTI-induced immune-escape mutations, which might challenge the efficacy of HBV vaccination [27]. In our study, despite the frequent association of the primary resistance mutations M204V/I with the compensatory L180M, V173L, and L80V/I mutations, lamivudine-resistant HBV strains tend to be associated with lower viral loads compared to wild-type strains; further characterization of the viral strains, using Next-Generation Sequencing (NGS), might reveal the presence of other mutations that alter viral replication. For example, the A181T mutation, also common in this study’s patients, was associated with a stop codon in the overlapping portion of the S gene that alters the virion, yet not the subviral particles’ secretion [28].

The recently diagnosed IDUs were more frequently infected with genotype D, and presented RAMs in 63.5% of the cases, despite being treatment naïve, suggesting transmitted drug resistance and the probable acquisition of HBV infection in large transnational networks of previously treated IDUs. In the same way, phylogenetic studies on the HIV-infecting subtypes in IDUs in Romania revealed small transmission clusters, with other genotypes than those encountered locally [29].

In our study, IDU co-infected patients tend to have a higher frequency of active liver disease, with hepatocytolysis and advanced liver fibrosis, indicating an urgent need for the initiation of HBV-active antiretroviral therapy, and possibly reflecting the significantly higher percentage of HDV co-infection in this category of patients as well. Worldwide cross-sectional studies indicate that the prevalence of HDV infection among HIV–HBV co-infected patients ranges from 1.2 to 25% [30]; in this study, the prevalence was 16.7%. Liver-related mortality is substantially higher in HIV–HBV co-infected patients compared to HIV- or HBV mono-infected patients, but can be decreased after the introduction of tenofovir-containing ART therapy [31,32,33], which is recommended as the first line of treatment in HIV–HBV co-infected patients. Hepatitis delta treatment, limited to pegylated interferon-alpha for a long time, with a sustained virologic response lower than 25% [18,34], is now experiencing a series of landscape changes, with the recent approval of bulevirtide—the hepatic NTCP (sodium/taurocholate co-transporting polypeptide) receptor inhibitor [35], and the discovery of lonafarnib—the farnesyltransferase inhibitor that prevents prenylation of the large HDAg [36].

The presence of resistance mutations is an important factor in future treatment response. A relatively high prevalence of the N236T mutation was recorded in the study patients, which is frequently associated with the A181T/V mutation, a combination that confers decreased efficacy of tenofovir in vitro and was linked to a delayed response to TDF/TAF in patients with high viral loads [37], or with the compensatory L80V mutation, reported to be associated with LAM and ADV resistance.

## 5. Conclusions

In Romanian HIV–HBV co-infected patients, HBV genotype A is mainly detected in long-term survivors infected during childhood, who present extensive resistance to LAM, but have no signs of advanced liver fibrosis. The recently diagnosed IDUs are more frequently infected with genotype D, and present resistance mutations in more than half of the cases, despite being treatment naïve. Further monitoring of the newly diagnosed HBV mono-infected and co-infected patients is needed, in order to evaluate the presence of transmitted drug resistance and its influence on the response to treatment.

## Figures and Tables

**Table 1:** Patients’ characteristics.

	Long-Term HIV Cohort *N* = 81	IDUs *N* = 36	*p*
**Age (years)**	27.55 ± 0.7908	30 ± 0.9909	0.0575
**Male (%)**	62.7%	65.9%	0.892
**Mean HIV viral load** **(log10 copies/mL)**	4 ± 3.6	5.5 ± 5.3	**0.0001**
**Undetectable HIV viral load (%)**	30.8%	5.6%	**0.0027**
**CD4 (cells/mm^3^) mean**	533.6 ± 327.7	511.9 ± 315.4	0.626
**CD4 < 200 cells/mL (%)**	18.52%	25%	0.4229
**Mean HBV viral load** **(log10 IU/mL)**	6.99 ± 6.5	6.9 ± 6.5	0.978
**Undetectable HBV viral load (%)**	33.3%	11.1%	**0.0119**
**HBV viral load > 1000 IU/mL (%)**	32.1%	52.8%	**0.0338**
**HBeAg positive (%)**	11.1%	50.00%	**0.0172**
**HDV co-infection (%)**	2.5%	16.7%	**0.005**
**APRI**	0.57 ± 0.092	4.9 ± 2.189	**0.004**
**APRI > 1 (%)**	12.3%	36.1%	**0.0339**

HIV, human immunodeficiency virus; HBV, hepatitis B virus; HBeAg, hepatitis Be antigen; HDV, hepatitis D virus; APRI, AST-to-Platelet Ratio Index.

**Table 2:** Patients’ characteristics by HBV infecting genotype.

	HBV Genotype A *N* = 30	HBV Genotype D *N* = 15	*p*
**Mean HIV viral load** **(log10 copies/mL)**	4.9 ± 4.3	4.99 ± 4.4	0.4110
**Undetectable HIV viral load (%)**	30%	0%	**0.0177**
**Mean CD4 (cells/mm^3^)**	557.6 ± 75.65	511.7 ± 136.4	0.7567
**CD4 < 200 cells/mL (%)**	16.67%	6.67%	0.3522
**Mean HBV viral load** **(log10 IU/mL)**	6.99 ± 6.6	6.88 ± 6.5	0.2488
**HBe Ag positive (%)**	33.33%	20.00%	0.3522
**APRI score mean**	2.053 ± 1.024	0.5163 ± 0.09038	0.238
**APRI > 1 (%)**	16.6%	13.3%	0.2049

**Table 3:** Frequency of individual RAMs.

RAM	HBV Genotype A *N* = 30	HBV Genotype D *N* = 15	*p*
**M204V/I**	53.3%	40%	0.399
**L180M**	36.6%	26.6%	0.8257
**V173L**	50%	40%	0.3933
**L80V**	40%	6.7%	**0.032**
**N236T**	43.3%	0%	**0.004**
**A181T/V**	16.6%	13.3%	0.8257
**Wild type**	13.3%	20%	0.5608

M204V/I; L180M; V173L; L80V; N236T; A181T/V, HBV resistance-associated mutations.

**Table 4:** HBV drug resistance pattern among 45 HIV–HBV co-infected genotyped patients.

Pattern	Resistance to	Number	Percent
L180M, M204V/I	LAM	22	48.8%
V173L, L180M, M204V/I	LAM	23	51.1%
L180M, M204V/I ± V173L	ETV	22	48.8%
N236T + A181T/V	ADV and reduced TDF/TAF	19	42.2%

LAM, lamivudine; ETV, entecavir; ADV, adefovir; TDF/TAF, tenofovir.

## Data Availability

Not applicable.
